# Agronomic solutions to decrease arsenic concentrations in rice

**DOI:** 10.1007/s10653-025-02508-7

**Published:** 2025-05-16

**Authors:** Marguerita E. Leavitt, Michele L. Reba, Angelia L. Seyfferth, Benjamin R. K. Runkle

**Affiliations:** 1 Department of Biological & Agricultural Engineering, University of Arkansas, Fayetteville, AR 72701 USA; 2 USDA-ARS Delta Water Management Research Unit, Jonesboro, AR 72401 USA; 3 Department of Plant and Soil Sciences, University of Delaware, Newark, DE 19716 USA

**Keywords:** Arsenic toxicity, Rice, Soil amendments, Alternate wetting and drying

## Abstract

Rice plants accumulate arsenic (As), a toxic metalloid found both naturally and as a form of anthropogenic pollution in rice fields. Arsenic concentrations in rice grain may pose human health risks, particularly when consumed regularly or used in food products for infants and young children. The purpose of this review is to summarize evidence-based mitigation strategies for minimizing the As content of rice and establish recommendations for their implementation. Mitigation strategies include irrigation management practices that introduce aerobic periods, applying soil and foliar amendments that decrease As plant-uptake, selecting and developing cultivars with lower rates of As-uptake, and post-harvest processing. In addition to a literature review, we interviewed rice producers and stakeholders to identify barriers or knowledge gaps to implementing the mitigation strategies. Alternate wetting and drying irrigation showed high effectiveness in decreasing grain As concentrations and is also climate-smart and can be cost-neutral. Combining irrigation management with select amendments maximized the decrease of grain As concentrations. Combining treatments will allow a choice of options to accommodate different farm circumstances, though nearly all field-level treatments lack experimental evidence from trials at production-scale operations (i.e., > 1 ha, with commercial management). Thus, more research is needed to develop best management strategies at the field scale in collaboration with farmers.

**Supplementary Information:**

The online version contains supplementary material available at 10.1007/s10653-025-02508-7.

## Introduction

Arsenic (As) is a toxic metalloid found in soil and water that can pose a human health risk upon intake into the body through ingested food or water containing arsenic. In the environment, arsenic is generally found as the inorganic As (iAs) compounds arsenite (iAs(III) or H_3_AsO_3_ at circumneutral pH) and arsenate (iAs(V) or H_2_AsO_4_^−^ or HAsO_4_^2−^ at circumneutral pH) and as organic As compounds (oAs) including dimethylarsinic acid (DMA) and monomethylarsonic acid (MMA). While iAs is more acutely toxic to humans and had been considered more of a concern than oAs compounds (Zhao et al., 2010a, 2010b), both iAs and oAs compounds like DMA are carcinogens (IARC, 2012), and some newly discovered oAs compounds, such as dimethylmonothioarsenate (DMMTA) are more toxic than iAs (Dai et al., 2022; Planer-Friedrich et al., 2022). Rice is the main dietary source of iAs for adults in cultures where rice makes up a large portion of the diet (ASTDR, 2007; Meharg et al., 2008) and is the primary dietary source of iAs for infants and young children, whose first solid food is often rice cereal. Most governments have set limits on the amount of iAs that can be found in rice and be considered safe for consumption. In China this limit is set at 0.15 mg kg^−1^, whereas the EU sets the limit at 0.2 mg kg^−1^ (EU, 2015; USDA, 2006). Both the EU and the US recommend no more than 0.1 mg kg^−1^ of iAs in rice intended for baby food (EU, 2015; US-FDA, 2016). However, the median level of iAs in white rice sold worldwide reached or exceeded the limit of 0.1 mg kg^−1^ for 36 of 63 samples tested (Meharg et al., 2009), so the mitigation of rice uptake of As in the grain is critical.

Rice accumulates more As in the grain than other cereals such as wheat and barley for two main reasons. First, As is mobilized in soil during the flooded, anaerobic conditions under which rice is commonly grown and is further reduced to arsenite by iron- and arsenic-reducing bacteria (Dai et al., 2020; Jia et al., 2014). Second, the uptake of arsenite by rice follows the Si uptake pathway, which is highly efficient and unique to rice (Ma et al., 2008; Williams et al., 2007a, 2007b; Zhao et al., 2010a, 2010b). These molecular mechanisms and the phytotoxicity of As on rice plants, and a mix of management and biotechnological solutions have been reviewed recently (Geng et al., 2024).

Indeed, there are on-farm management practices and post-harvest processing methods that can decrease As concentrations in rice grain and/or in the marketed product. The purpose of this review is to synthesize the evidence basis for mitigation practices of As in rice, their efficacy and potential drawbacks, and the conditions under which their use should be recommended. Uniquely, we also interviewed rice producers, millers, and supply-chain partners to identify barriers to implementation and to further devise solutions. We start with a brief review of how arsenic accumulates in rice.

## Arsenic sources and accumulation in rice

Compounds containing As occur naturally in the environment from the weathering of As-bearing minerals and emissions from volcanoes and hot springs but can also be caused by anthropogenic contamination. These sources include mining, smelting, and agricultural activities that release As into the soil, water, and air (ASTDR, 2007; Zhao et al., 2010a, 2010b) or the historic application of organic As (oAs) compounds as pesticides and herbicides for cotton and fruit tree production (ASTDR, 2007; Zhao et al., 2010a, 2010b). It has been suggested that elevated levels of grain As in rice grown in the Mid-South US relative to rice grown in California, Europe, and parts of Asia may be due to residual As from pesticide and herbicide remaining in the soil (Williams et al., 2005; Williams et al., 2007), but this link has not been directly shown.

In the rice plant, roots contain the highest level of As, followed by the stem, leaves, husk, and grain (Moulick et al., 2018; Pan et al., 2022). The amount of total As and proportion of different As species found in rice grain varies by location, method of production, and other biogeochemical factors (Suriyagoda et al., 2018). In general, rice contains higher levels of iAs than oAs, with iAs(III) predominating in rice grown under anaerobic soil conditions and iAs(V) predominating in rice grown under aerobic soil conditions (Zhao et al., 2010a, 2010b). Methylated species like DMA, DMMTA, and MMA usually make up less than 50% of total grain As, though oAs can also exceed 60% of total As (Colina Blanco et al., 2021; Meharg et al., 2008; Sommella et al., 2013; Williams et al., 2005).

Rice is typically grown under flooded conditions that cause the soil environment to become anoxic, supporting anaerobic microbial metabolisms that promote As mobilization and plant uptake. These conditions promote the reductive dissolution of As-containing Fe oxide minerals in soil, leading to As release, and favor the reduction of iAs(V) to the more mobile iAs(III) (Takahashi et al., 2004). Though much of the As is taken up as iAs(III), some iAs(V) is still taken up under anaerobic conditions because the root aerenchyma release oxygen that allows iAs(III) to oxidize to iAs(V) (Wu et al., 2012). Inorganic As(III) is taken up by the highly efficient Si transporters *Lsi1* and *Lsi2* while iAs(V) is taken up by phosphate transporters, though the exact transporters have not been identified (Abedin et al., 2002; Ma et al., 2008). There is evidence that DMA and MMA are also taken up by *Lsi1* transporters (Limmer et al., 2018a, 2018b) but uptake by these transporters is insufficient to account for all oAs uptake, which suggests that there are additional unknown uptake mechanisms (Li et al., 2009a, 2009b). Because As is also phytotoxic, rice plants have evolved methods for limiting As transport such as complexing iAs(III) with phytochelatins and storing it in vacuoles or downregulating phosphate transporters under high As stress (Begum et al., 2016).

The oxidization of the area around the aerenchyma also encourages the formation of Fe plaques that can adsorb As species, limiting uptake into the plant (Chen et al., 1980; Hossain et al., 2009; Seyfferth et al., 2010). The species and amount of As adsorbed by Fe plaques depends on complex interactions with soil pH and the form of the Fe oxide in the plaque (Dixit & Hering, 2003; Seyfferth et al., 2019). Species of oAs are less easily sorbed to Fe oxides than species of iAs and are more plant-available (Li et al., 2009a, 2009b), though oAs species usually make up a smaller percentage of total As than iAs species in the soil and therefore in the plant (Chowdhury et al., 2020; Honma et al., 2016; Lin et al., 2018).

## Literature review and stakeholder interviews

To identify arsenic mitigation opportunities, we performed a literature search using the University of Arkansas library database and spanned 2008 through 2024. Ultimately, this review encompasses 120 studies across 14 countries and consists of 44 field studies, 68 pot studies, and 8 studies on postharvest practices. Studies that focused only on root or shoot As but not grain As were excluded unless they contributed something unique to other studies; these studies are noted in the analysis below.

To determine the feasibility of mitigation options for limiting As intake associated with rice food products, we performed interviews with stakeholders in the rice production sector. These participants included people involved in agricultural processing, food production, the supply chain, and related areas in both the public and private sectors, whose activities may directly impact the commercial production of rice. We recruited participants from our networks, suggested additional contacts from the interview subjects, and opportunistically, e.g., at rice sector conferences. Our team asked questions (Table 1) designed to learn about current awareness of As in rice grain, any current activity to manage grain As levels, and the feasibility and estimated cost of implementing mitigation activities.

**Table 1:** Rice stakeholder interview questions

1	Who are you, and what is your professional relationship to the food and agriculture sector?
2	How would you rate your concern regarding arsenic in the rice grain you produce?
3	What within your production system would you be willing to modify to reduce arsenic in rice grains?
4	What steps are you currently taking to mitigate arsenic contamination in your product?
5	What would it take for you to implement practices that are known to reduce arsenic?
6	If implemented, what type of compensation would you require to implement these practices?
7	If implemented, how would it impact your operation-i.e., time, people, cost?
8	Do you have two to five suggested contacts of other people we could interview?

## Mitigation strategies

### Cultivar selection

Rice uptake of As can be limited by selecting cultivars that accumulate less As in the grain. Though plant As availability is largely dependent on environmental factors, As uptake and grain accumulation are significantly affected by cultivar (Farrow et al., 2015; Islam et al., 2017; Norton et al., 2009, 2012). Appropriate cultivar selection can lower grain total As across a broad range from 4 to 98% (Jiang et al., 2012; Pillai et al., 2010). Some cultivars limit As uptake through root transporters (Lu et al., 2010; Ma et al., 2008; Yang et al., 2021) or limit transport from the roots to the grain (Heuschele et al., 2017; Hu et al., 2013; Rahman et al., 2007; Roy et al., 2023). Other cultivars have root characteristics that encourage As sequestration on the root surface via adsorption or coprecipitation with iron plaque (Pan et al., 2014; Wu et al., 2015) or growth patterns that limit As uptake during grain-filling (Norton et al., 2012; Pillai et al., 2010). Some cultivars store more As in the husk or the root (Rahman et al., 2007; Roy et al., 2023), while other cultivars store more As in shoot or leaf tissue (Heuschele et al., 2017). Commercial rice cultivars have a relatively rapid turnover rate to changing agronomic conditions, which complicates consistent analysis of their environmental impact (Atlin et al., 2017; Linquist et al., 2018), so routine screening of new cultivars for As uptake should be implemented for cultivar selection to be a viable method of limiting As concentrations in commercial rice.

The timing and duration of cultivar growth stages may also influence grain As concentrations and speciation, though more research is needed in this area. Studies have argued both that later heading and flowering had higher As grain As (Norton et al., 2012; Pillai et al., 2010) and that early-heading cultivars had higher grain As (Duan et al., 2017). Additionally, for As speciation in the grain some studies found that only iAs varied by cultivar (Chi et al., 2018; Norton et al., 2012; Pan et al., 2014), while others found both iAs and oAs varied (Norton et al., 2009; Pillai et al., 2010). Cultivars of different rice subspecies also vary with respect to grain As concentration. *Japonica* cultivars generally had lower grain As concentrations than *Indica* cultivars (Carracelas et al., 2019; Heuschele et al., 2017; Jiang et al., 2012; Norton et al., 2012; Seyfferth & Fendorf, 2012). However, at least one study found the opposite, that *Japonica* cultivars accumulated more total grain As than *Indica* cultivars (Chi et al., 2018). The studies supporting the higher As accumulation potential of *Indica* compared to *Japonica* examined more than 500 cultivars, while the study supporting the higher As accumulation potential of *Japonica* examined 51 cultivars. This difference in sample size suggests that *Indica,* in general, has a higher tendency to accumulate grain As than *Japonica*, but that differences in accumulation for individual varieties means some *Japonica* cultivars have higher grain total As than some *Indica* cultivars. The difference in As accumulation between subspecies relates to yield characteristics, as high-yielding cultivars have higher total As accumulation than lower-yielding cultivars (Chi et al., 2018; Samal et al., 2021; Sandhi et al., 2017).

Genetic mapping can be used to identify genes linked to As uptake, which enables breeders to screen cultivars for potential low-As accumulators or to engineer new cultivars with low As accumulation (Fernández-Baca et al., 2021; Murugaiyan et al., 2019; Song et al., 2014). The genetic basis for As uptake is not fully understood, but several potential genes and mechanisms have been identified. The *OsABCC1* gene was upregulated in response to high levels of As and knocking out the gene led to lower As tolerance (Song et al., 2014). Genetically engineering plants to overexpress *OsABCC1* resulted in lower rates of As translocation from root to shoot and internode to grain (Deng et al., 2018), likely via vacuole storage (Song et al., 2014). Rice plants with mutations of the iAs(III) *Lsi1* and *Lsi2* transporter genes had significantly lower total grain As than wild-type rice, as well as lower uptake into the root and transport within the plant (Ma et al., 2008). However, selecting or engineering cultivars with low expression of these transporters is unlikely to be a viable method of finding a low-As cultivar because *Lsi1* and *Lsi2* are primarily Si transporters and rice plants need Si for optimum growth (Ma et al., 2008). It might be more useful to target the phosphate uptake pathway, which is also an uptake pathway for iAs(V) (Abedin et al., 2002), but this species is typically in low abundance in most rice paddies. Indeed, some low-As cultivars had higher rates of P uptake that are generally beneficial to the plant (Lu et al., 2010; Yang et al., 2021).

Breeding or engineering low-As rice cultivars is challenging because cultivars that accumulate low amounts of As tend to accumulate high amounts of Cd even under the same redox conditions (Duan et al., 2017; Hu et al., 2013), though cultivars with relatively low accumulation of both metals do exist (Chi et al., 2018). Differences in As accumulation by cultivar were also not always the same at different sites and during different years, indicating strong interaction with climate and management practices (Norton et al., 2009, 2012; Pillai et al., 2010; Rahman et al., 2007). Therefore, breeding for low-As cultivars may need to be site- and practice-specific (Norton et al., 2009).

### Irrigation management

Of all the mitigation methods surveyed, water management techniques are perhaps the most effective in limiting As concentrations in rice. Though rice has traditionally been grown under continuously flooded conditions (Singh et al., 2017), the need to improve water-use efficiency, and concern over high levels of methane (CH_4_), a potent greenhouse gas emitted during periods of extended flooding (Banker et al., 1995; Saunois et al., 2020) has led to irrigation management that introduces aerobic periods during the growing season. These irrigation management strategies include sprinkler irrigation, alternate wetting and drying (AWD) (also sometimes called intermittent flooding), furrow irrigation, aerobic cultivation, and rainfed irrigation. Sprinkler irrigation uses an overhead sprinkler to water the top of the soil periodically but does not fully saturate the field (Vories et al., 2013). AWD involves flooding the field and then allowing it to dry down to a specified moisture level before reflooding (Lampayan et al., 2015), and this cycle can be repeated multiple times throughout the growth period. In furrow irrigation, water is moved down furrows, and field drains are often, but not always, blocked to hold water at the bottom of fields. In aerobic cultivation, the field is never fully saturated but is kept watered to near-saturation (Bouman et al., 2007), and in rainfed cultivation, the crop is watered solely via precipitation (O’toole, 1982).

Despite differences in soil type, climate, and type of irrigation method applied, every study in this review found that irrigation methods with aerobic periods lowered total grain As anywhere from 10 to 98% in rice when compared to continuous flooding (Abu-Ali et al., 2023; Alvarenga et al., 2022; Arao et al., 2009; Carrijo et al., 2018; Chou et al., 2016; Codling & Chen, 2017; Fernández-Baca et al., 2021; Honma et al., 2016a, 2016b; Hu et al., 2013; Islam et al., 2017, 2020; Li et al., 2019, 2009a, 2009b; Limmer & Seyfferth, 2024; Linam et al., 2022; Ma et al., 2014; Mukherjee et al., 2017; Norton et al., 2013; Orasen et al., 2019; Sengupta et al., 2021; Seyfferth et al., 2019a, 2019b; Somenahally et al., 2011a, 2011b; Somenahally et al., 2011a, 2011b; Spanu et al., 2012, 2020; Talukder et al., 2012; Wu et al., 2021; Xu et al., 2008; Yang et al., 2023). The creation of aerobic conditions during non-inundated periods oxygenates the soil, raising the soil redox potential and oxidizes ferrous iron into ferric (oxyhydr)oxide minerals that adsorb iAs(III) and iAs(V), rendering them immobile (Takahashi et al., 2004). Because oAs compounds are produced during reduced conditions, this technique also decreases the production of oAs compounds in soil and thus uptake into the grain. Irrigation methods that include aerobic periods have the additional benefits of reducing water use by 18–74% in comparison to continuous flooding (Kahlown et al., 2007; Kato et al., 2009; Linquist et al., 2015; Spanu et al., 2012) and lowering emissions of CH_4_ by 39–83% (Leavitt et al., 2023; Linquist et al., 2018; Runkle et al., 2019). Additionally, in many conditions, AWD implementation can be cost-neutral or even cost-effective, as it relies on lower water inputs (Nalley et al., 2015).

In general, the effect of lowering As mobilization with irrigation management was more pronounced with treatments that had longer periods of time under aerobic management. The most extreme of these was with rice grown entirely under aerobic conditions or under aerobic sprinkler irrigation, which resulted in the greatest reduction in total grain As, up to 98% lower than continuous flooding (Spanu et al., 2012). Rice grown under forms of AWD with longer and more frequent drying events had higher reductions in total grain As than forms of AWD with shorter or less frequent drying events (Arao et al., 2009; Carrijo et al., 2018; Fernández-Baca et al., 2021; Honma et al., 2016a, 2016b; Hu et al., 2013; Li et al., 2019, 2009a, 2009b; Limmer & Seyfferth, 2024; Linam et al., 2022; Sengupta et al., 2021; Spanu et al., 2020; Wu et al., 2021; Yang et al., 2023). Safe-AWD, a conservative form of AWD in which the field is reflooded when the water table reaches 15 cm below the soil surface, did not significantly lower total grain As in most studies (Carrijo et al., 2018; Fernández-Baca et al., 2021; Li et al., 2019), though it did manage to lower it by 15–33% in one study (S. Islam et al., 2017; Limmer & Seyfferth, 2024). This difference may be driven by dry down frequency or soil texture and drainage conditions (described in Carrijo et al., 2018) when Safe-AWD did not sufficiently dry the soil, thus never achieving fully aerobic conditions (Islam et al., 2017).

Aerobic irrigation typically lowered both iAs and oAs 5–90% in the grain (Honma et al., 2016; Honma et al., 2016a, 2016b; S. Islam et al., 2017; Li et al., 2019; Limmer & Seyfferth, 2024; Linam et al., 2022; R. Ma et al., 2014; Norton et al., 2013; Seyfferth et al., 2019a, 2019b). In addition, some studies found that the ratio of iAs to oAs decreased under aerobic irrigation (Chou et al., 2016; Codling & Chen, 2017), while others found that it increased (Li et al., 2009a, 2009b; Somenahally et al., 2011a, 2011b; Xu et al., 2008). Notably, in studies where aerobic irrigation increased the ratio of iAs to oAs in the grain, total iAs was still 5–66% lower for rice under aerobic irrigation than for rice under continuous flooding (Li et al., 2009a, 2009b; Somenahally et al., 2011a, 2011b; Xu et al., 2008). The more aerobic conditions decrease the mobilization of iAs and thus provoke less uptake into the plant and lower the iAs available for methylation by microbes.

One drawback of employing irrigation methods with aerobic periods is that they can result in higher grain Cd concentrations in some soils because Cd is most bioavailable under aerobic conditions (Arao et al., 2009). Cd compounds in acidic soils frequently occur as CdS, and when the sulfide component of CdS is oxidized to sulfate, Cd is mobilized (Arao et al., 2009; Seyfferth et al., 2019a, 2019b). The levels of Cd in the rice grain increased under aerobic irrigation for 12 of the 31 studies in this review (Alvarenga et al., 2022; Arao et al., 2009; Honma et al., 2016a, 2016b; Hu et al., 2013; Li et al., 2019; Limmer & Seyfferth, 2024; Linam et al., 2022; Orasen et al., 2019; Seyfferth et al., 2019a, 2019b; Spanu et al., 2020; D. Yang et al., 2023), with three of the studies measuring Cd that exceeded the recommended limit of 0.4 mg kg^−1^ established by the Food and Agriculture Organization (FAO) of the United Nations (Arao et al., 2009; FAO-WHO Codex Alimentarius, 2019; Honma et al., 2016; Hu et al., 2013). Instances in which Cd exceeded the FAO limit occurred under irrigation treatments that maintained aerobic conditions for the majority of the season (Arao et al., 2009; Hu et al., 2013) or relied solely on precipitation (Honma et al., 2016). Less severe forms of aerobic irrigation were able to lower grain As concentrations without increasing Cd beyond allowable limits (Alvarenga et al., 2022; Arao et al., 2009; Honma et al., 2016a, 2016b; C. Li et al., 2019; Linam et al., 2022; Orasen et al., 2019; Seyfferth et al., 2019a, 2019b; Spanu et al., 2020). It is thus crucial to ensure that Cd is not elevated when attempting to lower grain As concentrations through irrigation management.

Yield loss is also sometimes associated with irrigation methods that introduce aerobic periods. Yield loss for studies in this review ranged from 3 to 44% compared to crops managed under continuous flooding (Arao et al., 2009; Carracelas et al., 2019; Chou et al., 2016; Fernández-Baca et al., 2021; Honma et al., 2016a, 2016b; Islam et al., 2020; Mukherjee et al., 2017; Seyfferth et al., 2019a, 2019b), with higher yield loss associated with higher degrees of drying such as fully aerobic treatments and severe AWD (Arao et al., 2009; Carracelas et al., 2019; Chou et al., 2016; Fernández-Baca et al., 2021; Honma et al., 2016a, 2016b; Mukherjee et al., 2017). Contrarily, other studies found that irrigation methods with aerobic periods had no effect on yield () or even increased yield (Hu et al., 2013; Islam et al., 2017; Linam et al., 2022; Xu et al., 2008) compared to continuous flooding. The effect also depends on cultivar, with AWD causing a 15–32% yield decrease in some cultivars and a yield increase or no effect in others (Hu et al., 2013; Orasen et al., 2019). The effect also depends on timing, as drying events during panicle differentiation caused yield loss, while drying events of similar severity during other parts of the season had no effect on yield (Orasen et al., 2019). Given the number of studies that did not demonstrate yield loss, it is probable that the degree of drying during aerobic irrigation could be refined in some cases to determine a point where irrigation management is sufficient to decrease grain As with a low risk of yield loss (Carrijo et al., 2017, 2022).

Each method of irrigation with aerobic periods has its own benefits and drawbacks. Sprinkler irrigation was able to reduce total grain As by 69–98% compared to continuous flooding without raising grain Cd beyond FAO limits (Alvarenga et al., 2022; FAO-WHO Codex Alimentarius, 2019; Spanu et al., 2012, 2020). It can also be implemented on fields that are too uneven for continuous flooding or AWD (Spanu et al., 2012), but it has high equipment costs, requires energy use (Tracy et al., 1993), and is not appropriate for all soil types (Hardke et al., 2018). Rainfed rice can decrease total grain As by 72% compared to continuous flooding (Honma et al., ), but is only viable in climates with high and predictable precipitation. AWD can decrease total grain As by 10–92% depending on the number and length of drying periods (Arao et al., 2009; Carrijo et al., 2018; Chou et al., 2016; Honma et al., 2016a, 2016b; Islam et al., 2017, 2020; Li et al., 2019; Linam et al., 2022; Mukherjee et al., 2017; Orasen et al., 2019; Sengupta et al., 2021; Somenahally et al., 2011a, 2011b; Somenahally et al., 2011a, 2011b). AWD is applicable to any field with silt loam or clay textured soil (Hardke et al., 2018), and it can reduce water use by up to 38% (Carrijo et al., 2017; Lampayan et al., 2015). To be more effective, it can benefit from either zero-grading the field which is a high start-up cost (Watkins et al., 2007) or applying multiple inlet irrigation (Hardke, 2018), and it requires tight control over water supply and availability. Fully or mostly aerobic cultivation reduced total grain As 15–100% (Chou et al., 2016; Codling & Chen, 2017; Hu et al., 2013; Li et al., 2009a, 2009b; Linam et al., 2022; Mukherjee et al., 2017; Norton et al., 2013; Seyfferth et al., 2019a, 2019b; Wu et al., 2021; Xu et al., 2008) and is highly water-use efficient (Kato et al., 2009), but had the highest grain Cd accumulation (Hu et al., 2013) and was the most prone to yield loss compared to other methods in this review (Chou et al., 2016; Hu et al., 2013; Islam et al., 2020; Seyfferth et al., 2019a, 2019b). Furrow irrigation lowered total grain As by 50% in the one study in this review using this technique (Spanu et al., 2020). Furrow irrigation is versatile in that it can be easily rotated with crops other than rice and works well in soils that lack the clay content needed to sustain continuous flooding, but like most aerobic irrigation methods may increase the weed burden and risk lower yield (Tracy et al., 1993). No studies to date have investigated the Cd content of furrow-irrigated rice.

Due to the high effectiveness of irrigation methods that include aerobic periods in lowering total grain As, we recommend them as good alternatives to traditional flooding for any field that does not also have relatively high levels of soil Cd and does not have other risk factors for yield loss such as a high weed burden or potential for disease. In cases of high soil Cd, aerobic irrigation should be used alongside amendments that decrease plant Cd accumulation. The irrigation method should be chosen to suit the needs of the producer given their field conditions. Further research is necessary to determine an ideal moisture balance for lowering As without also lowering yield in different soil types and climates.

### Amendments

**Fig. 1:**
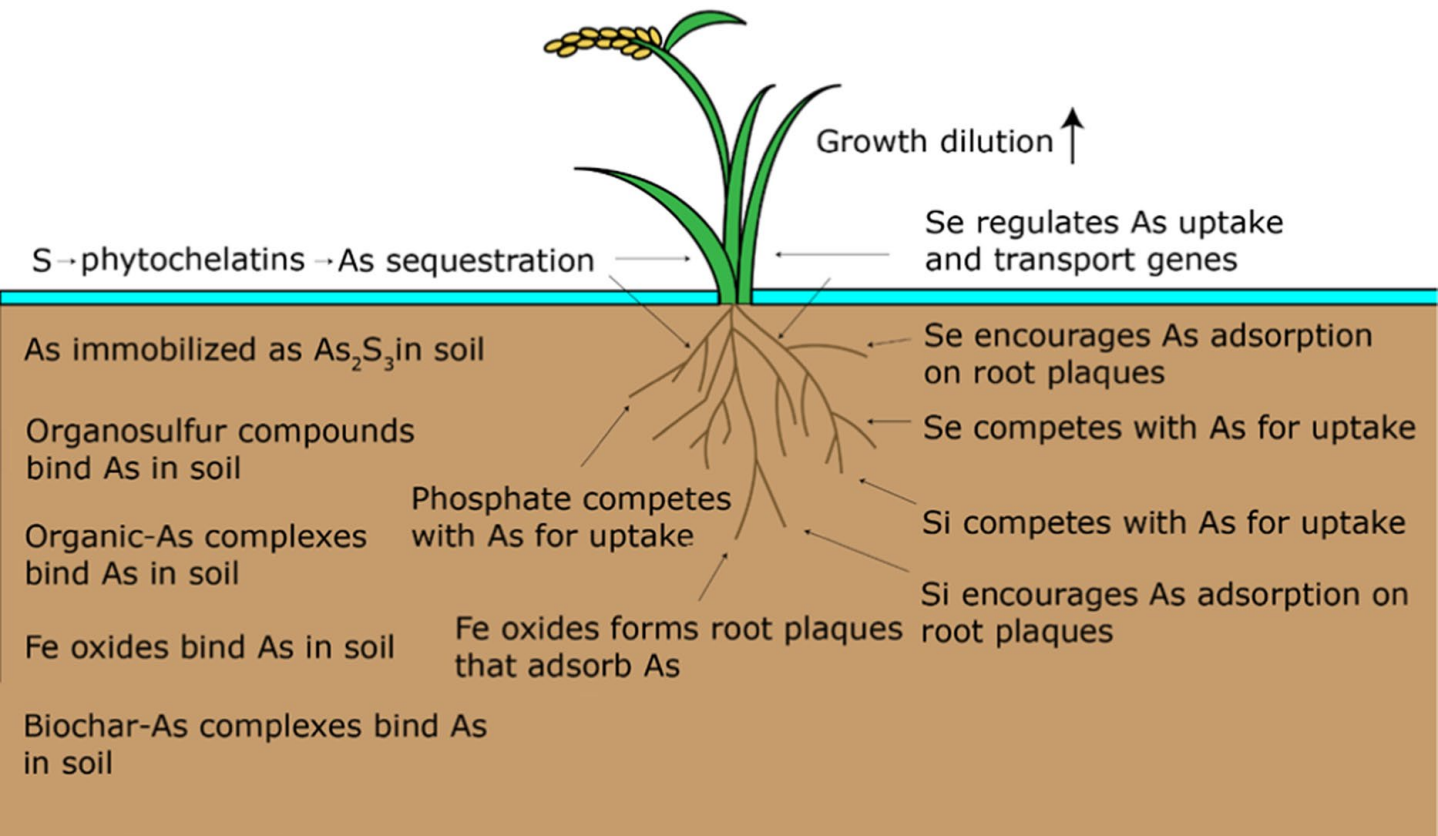
Overview of the mechanism of action for each amendment in the soil and in the plant

The application of soil amendments or foliar sprays to decrease As concentrations in rice grain has been extensively studied. Crop amendments act by binding or adsorbing As oxyanions in the soil or porewater, by reacting with As oxyanions in a way that converts them to a less mobile form, by competing with As for uptake in the plant (F.-J. Zhao et al., 2010a, 2010b) which enhances growth and creates growth dilution, or by preventing As transport to the grain from within the plant (Fig. 1). They can also stimulate the growth of microorganisms that deter As mobility or uptake (Bakhat et al., 2017). These amendments can be organic, such as compost or biochar, or inorganic, such as Si, Se, Fe, S, and P compounds, and vary in cost, availability, and effectiveness. Amendments are usually applied to the soil prior to planting or, less commonly, sprayed on the foliage.

#### Silicon

Soil and foliar amendments containing high proportions of Si compounds have been demonstrated to lower As uptake in plants (for a detailed review, see Seyfferth et al., 2018). Si is an important nutrient for rice that improves disease resistance and alleviate plant stress, resulting in stronger plants increased yield and biomass (Savant et al., 1996). They can also lower grain Cd concentrations by inhibiting Cd transport from shoot to grain, by sequestering it in the cell walls and by down-regulating genes involved in Cd transport, or promoting growth dilution (C. Guo et al., 2018; Liu et al., 2009; Seyfferth et al., 2019a, 2019b; Shao et al., 2017). Si amendments come in many forms, including the Si-rich residue of rice straw and rice husk, ash or char made from rice straw and rice husk, silicic acid, calcium silicate, silica gel, silica nanoparticles, diatomaceous earth, potash, and sodium silicate. Each form of Si has different effectiveness due to different rates of Si dissolution and modes of action (Seyfferth et al., 2018).

Some of the reduction in As following Si application can be attributed to growth dilution (Fleck et al., 2013), but Si amendments also encourage As sequestration in iron plaque and inhibit As uptake through the downregulation of Si transporters and uptake competition (Seyfferth et al., 2018). As the level of Si in the porewater increases, Si transporters are down-regulated and Si competes with iAs(III) for uptake, resulting in less As uptake at high levels of porewater Si (Pan et al., 2022; Seyfferth & Fendorf, 2012; Seyfferth et al., 2019a, 2019b). A pot study with different Si amendments also demonstrated how some yield loss caused by As was alleviated by Si, by creating more competition for uptake, more ferrihydrite on plaque, as well as growth dilution (Teasley et al., 2017). Si application also interacts with Fe plaques that form on plant roots and adsorb As. Si addition slows the crystallization of ferrihydrite into other forms, increasing the capacity of the plaque for As storage (Amaral et al., 2017; Limmer et al., 2022). Fe plaques composed of larger proportions of ferrihydrite relative to other species have a higher ability to adsorb As (Limmer et al., 2018a, 2018b; Teasley et al., 2017).

The majority of studies reviewed found that Si amendments decreased grain total As 7% to an estimated 78% at some level of application (Dwivedi et al., 2020; Fleck et al., 2013; G. Li et al., 2018; Limmer et al., 2018a, 2018b; Limmer & Seyfferth, 2022; Pan et al., 2022; Seyfferth et al., 2016; Seyfferth et al., 2019a, 2019b; Seyfferth & Fendorf, 2012; Teasley et al., 2017; Wang et al., 2016; Zhang et al., 2020), though some studies found that Si amendments lowered As levels in the shoot but not the grain (Dykes et al., 2021; Limmer et al., 2018a, 2018b; Linam et al., 2022). One study found that Si in the form of diatomaceous earth increased grain As, which was attributed to the competition between Si and As for adsorption on the soil particles (Seyfferth & Fendorf, 2012). Diatomaceous earth has low solubility and released only enough Si into the porewater to mobilize As but not enough to compete with As for uptake or to downregulate the transporters (Seyfferth & Fendorf, 2012). Another study found that rice straw application increased grain As due to the rice straw itself containing high levels of As (Wang et al., 2016) and likely because straw application promotes soil reducing conditions that mobilize As while husk application does not (Penido et al., 2016).

Experiments comparing forms of Si amendment found varied effectiveness. Some amendments such as calcium silicate release Si quickly, but may be ineffective when applied early in the season as their Si is released before plant demand for Si is high; amendments like husk release Si at a slow rate (Teasley et al., 2017). Studies comparing husk and husk ash and found that husk was more effective at lowering total grain As (Seyfferth et al., 2016; Teasley et al., 2017). The rate of application was important, with higher rates being more effective in general (H.-Y. Wang et al., 2016). The level of plant-available Si in different kinds of amendment varies, and amendments with lower available Si levels, such as calcium silicate slag, are ineffective at low rates (Matsumoto et al., 2015). In Si-deficient soil, low levels of Si amendments may be insufficient to raise porewater Si enough to downregulate transporters or compete with iAs(III) for uptake (Linam et al., 2022). While soil amendments were typically applied at the beginning of the season, foliar application timing had an impact, with effective reduction only occurring when Si was applied at tillering or jointing (internode elongation) (S. Zhang et al., 2020). One study found foliar application more effective than soil application but research in that area is limited (Dwivedi et al., 2020).

The majority of studies that measured grain As speciation found that Si application decreased grain iAs an estimated 18–62% (Fleck et al., 2013; Li et al., 2018; Limmer & Seyfferth, 2022; Limmer et al., 2018a, 2018b; Seyfferth et al., 2016, 2019b; Zhang et al., 2020) which is consistent with the competition of Si with iAs for uptake into the plant. The response of oAs to Si application was more complicated, with some studies finding Si amendments can lower grain oAs by 33–55% (Seyfferth et al., 2019a, 2019b; Teasley et al., 2017), while other studies found Si addition either had no effect on grain oAs (Fleck et al., 2013; Linam et al., 2022; Zhang et al., 2020) or raised it an estimated 40–69% (Li et al., 2018; Seyfferth et al., 2016). For studies with increased grain oAs it was speculated that Si application may have upregulated the *Lsi6* transporter that transports both Si and DMA into the panicle (Li et al., 2018). It is also possible that the Si in the soil promoted the conversion of soil iAs to oAs and therefore increased oAs uptake, particularly for organic-rich Si amendments like rice husk or straw that promote more reducing conditions, which are favorable for arsenic methylation (Dykes et al., 2021; Penido et al., 2016; Seyfferth et al., 2016).

It may also be useful to apply Si amendments in concert with AWD or aerobic irrigation. The combination of the two treatments has been shown to decrease grain total As to nondetectable levels, a higher decrease than under AWD or Si amendment alone (Seyfferth et al., 2019a, 2019b), though Linam et al., (2022) found that decreased grain As was driven solely by irrigation method. The response here may depend on the soil As concentration and the rate of Si application, as the rate of Si application and the soil As concentrations were higher under Seyfferth et al., (2019a, 2019b) than Linam et al., (2022). In summary, Si amendments can regulate grain As levels when applied in a sufficiently soluble form and at a high enough rate to effectively compete with As for uptake into the plant. Amendments in the form of rice husk or straw should be tested for As or other toxins before application (Wang et al., 2016).

#### Selenium

Selenium (Se) amendments have the potential to decrease As accumulation while also promoting growth and increasing yield (Lanza & Reis, 2021). In the form of either selenite (Se(IV)) or selenate (Se(VI)), Se has been used as a fertilizer when applied as a soil or foliar amendment or used as a substrate for seed priming prior to sowing. It plays a role in abating As stress by decreasing oxidative damage (Chauhan et al., 2020) and regulating genes associated with antioxidant response (Kumar et al., 2014). Selenium can lower grain As accumulation though the mechanism of action is not clear. It may compete with As for uptake by Si and phosphate transporters (Zhang et al., 2014; Zhao et al., 2010a, 2010b), regulate genes for uptake and transport within the plant (Chauhan et al., 2020), and encourage the production of glutathione and phytochelatins which are part of the process for vacuolar sequestration of As in plant tissue (Kumar et al., 2016). Selenium has also been shown to enhance As sequestration on Fe plaques on the root surface (Lv et al., 2020; Wan et al., 2018; Zhou et al., 2017).

All studies in this review that measured grain As found that Se application lowered grain total As an estimated 7–100% (Kaur et al., 2017; Lan et al., 2023; Liao et al., 2016; Lv et al., 2020; Moulick et al., 2018, 2023; Paniz et al., 2023; Pokhrel et al., 2020; Wan et al., 2018; Zhou et al., 2017), though the efficacy varied depending on the rate of application, speciation of Se, and the soil As content. In general higher application rates lowered grain total As (Liao et al., 2016; Lv et al., 2020; Zhou et al., 2017), though two studies found that the second-highest rate of application was more effective than the highest rate of application (Moulick et al., 2018, 2023), and one study found no difference in effectiveness between rates (Wan et al., 2018). Several studies found differences in effectiveness between Se(IV) and Se(VI), though the results were not consistent: some studies found Se(IV) was better at reducing grain total As or transfer of As from the root to aboveground parts of the plant (Camara et al., 2018; Hu et al., 2014; Liao et al., 2016) while others found that the relative effectiveness of Se(IV) and Se(VI) depended on the cultivar (Pokhrel et al., 2020). Amendments of Se may also be more effective when applied to soil with low to moderate levels of As rather than high levels of As (Moulick et al., 2018; Zhou et al., 2017). Studies reporting speciation of As in the grain were limited and contradictory, with one study finding Se application lowered both oAs and iAs (Lv et al., 2020) and another finding that Se application only affected iAs (Pokhrel et al., 2020).

Evidence for the effectiveness of Se application in reducing As accumulation is strong, but this amendment should be applied at low to moderate rates to avoid potential negative effects. Higher rates of application were generally more effective, but overdoses of Se can be toxic to the plant as well (Reis et al., 2020). And, while small amounts of Se are necessary for all living organisms, Se in large quantities is an environmental pollutant and can harm wildlife, particularly aquatic organisms and the birds that feed on them (U.S. EPA, 2018). Runoff from the irrigation of fields with high Se content is a major source of Se contamination (U.S. EPA, 2018). Furthermore, all studies on Se application in this review were pot studies, so extensive field-scale research on Se amendment strategies is recommended before promoting this method for reducing As accumulation in rice.

#### Iron

Soil amendments containing Fe compounds can immobilize soil As through adsorption and coprecipitation (S. Dixit & Hering, 2003; Ko et al., 2015). The majority of studies found that Fe amendments lowered total grain As with a range of effectiveness from 13 to 92% (Farrow et al., 2015; Ghosh et al., 2022; Honma et al., 2016; Hossain et al., 2009; Irem et al., 2019; Ko et al., 2015; Li et al., 2022a, 2022b; Matsumoto et al., 2015, 2016; Qiao et al., 2018; Yu et al., 2017a, 2017b; Zou et al., 2018), though not all forms of Fe were equally effective. When comparing Fe oxides to converter furnace slag, a high-Fe byproduct of steel production, the Fe oxides had a higher impact on total plant As because the slag had less exchangeable Fe content (Honma et al., 2016; Matsumoto et al., 2016). As with iron plaque, Fe oxides can strongly retain iAs and limit mobilization in soil. Fe oxides were more effective than ferrous sulfate (Li et al., 2022a, 2022b), not as effective as powdered zero-valent Fe (Matsumoto et al., 2015, 2016), and of similar effectiveness to ferrous chloride (Yu et al., 2017a, 2017b). The primary difference between amendments was iron content, with the zero-valent Fe powder being more than 90% Fe by weight, while the other forms were less than 60% (Matsumoto et al., 2015). The strength of the amendment’s effect on plant As increased as the amount of Fe increased. Most of the studies in this section of the review did not measure As speciation, but the ones that did found Fe amendments lowered both iAs and oAs in the grain (Honma et al., 2016; Matsumoto et al., 2016). Two studies found that Fe amendments did not lower total grain As (Lei et al., 2019; Liu et al., 2020). Of these, one applied Fe in the form of As-rich mine tailings (Lei et al., 2019) and the other applied Fe as lodestone (Liu et al., 2020), a mineral primarily made of the iron oxide magnetite (Cornell & Schwertmann, 2003). Magnetite has high Fe content (Cornell & Schwertmann, 2003), but is well crystalline, while the most effective Fe oxides reviewed were poorly crystalline forms with more reactive surface area (Matsumoto et al., 2015, 2016; Ultra et al., 2009; Yu et al., 2017a, 2017b).

With Fe amendments of the same form, higher rates of application tends to increase the effectiveness (Farrow et al., 2015; Zou et al., 2018). Despite this trend, one study found that lower rates of application (0.1%) decreased shoot As concentrations more than a higher rate (0.5%), suggesting the role of other factors (Ultra et al., 2009). Similarly, two successive studies compared the same amendments in different years, with the second year having double the amendment of the first year, but had similar decreases in grain As (Matsumoto et al., 2015, 2016), suggesting that there is a threshold beyond which additional Fe is not beneficial.

In general, most studies in this review support the supposition that Fe amendments are effective at reducing As uptake without causing additional problems, but the choice of amendment is key. Using Fe-rich mine wastes provides use for such materials, but drawbacks include the potential for contamination with the waste material. Even uncontaminated Fe amendments do not always have a beneficial effect. Fe can increase soil porewater As (Marin et al., 1992), and while Fe additions can encourage the formation of Fe plaques, under some conditions these plaques can act as As sources rather than sinks (Hossain et al., 2009). However, Fe amendments can improve plant growth (Ghosh et al., 2022; Irem et al., 2019; Li et al., 2022a, 2022b; Ultra et al., 2009; Yu et al., 2017a, 2017b; Zou et al., 2018), which may facilitate growth dilution. Further study is needed to mechanistically predict how Fe addition may lead to As release rather than sequestration, and to what extent, particularly at the field scale.

#### Sulfur

Recent studies have demonstrated that S compounds can limit the As accumulation in rice grain (Liu et al., 2021; Mridha et al., 2022; Zhang et al., 2021), though the mechanisms remain uncertain. Within soil, S can form organosulfur compounds that retain iAs(III) (Burton et al., 2014; Langner et al., 2012) and can also react directly with As to form As_2_S_3_ under reducing conditions (Hashimoto & Kanke, 2018). Some studies have found that S amendments promote the formation of Fe root plaques that can sequester As (Hu et al., 2007), though others have found they can discourage the formation of Fe plaques (Liu et al., 2021). Within the plant, S amendments improve resilience to stress because S is a vital component of glutathione, cysteine, and phytochelatins, which are chelating compounds that bind to As and sequester it in root and node vacuoles, detoxifying As and lowering grain As levels (Dixit et al., 2015; Song et al., 2014). There is evidence that S amendments also lower the translocation of As from the root to the aboveground parts of the plant, including grain (Mridha et al., 2022; Zhang et al., 2021, 2022).

The majority of reviewed studies found that S amendments lowered total grain As 27–72% in rice, though not under all management conditions (Liu et al., 2021; Meselhy et al., 2021; Mridha et al., 2022; Wisawapipat et al., 2021; Zhang et al., 2021). S amendments seem to be most effective at limiting grain As concentrations when used under flooded conditions, which allows the mobilized iAs(III) to react with S^2−^ and precipitate (Liu et al., 2021; Meselhy et al., 2021; Wisawapipat et al., 2021; Zhang et al., 2021). The magnitude of the effect was also rate dependent, with higher rates of S application resulting in lower grain As concentraitons (Mridha et al., 2022; Zhang et al., 2021). The type of S amendment has an impact on effectiveness, as nanoscale S was able to decrease total grain As while bulk S powder was not (Meselhy et al., 2021). However, studies comparing different forms of S amendment found no difference in grain As levels between them (Wisawapipat et al., 2021; Zhang et al., 2021), and it is not clear what properties make one form of S more effective than another, nor have the relative costs of the amendment options been considered.

Of the studies that measured As speciation, iAs followed a similar pattern to total As, with all studies finding that S application lowered grain iAs 25–79% (Fang et al., 2023; Liu et al., 2021; Wisawapipat et al., 2021; Zhang et al., 2022), but with the percent decrease being greatest for plants managed with continuous flooding (Fang et al., 2023; Wisawapipat et al., 2021). The effect of S amendments on grain oAs was less straightforward, with some studies finding that S amendment lowered oAs an estimated 26–60% (Liu et al., 2021; Zhang et al., 2022) and another finding it had no effect on oAs levels (Wisawapipat et al., 2021). One study found that S amendment raised grain oAs 63%, which was attributed to the amendment providing additional substrate for sulfate reducing bacteria that also methylate As (Fang et al., 2023). Increasing S has been associated with an increase in the formation of DMMTA under reducing conditions (Wang et al., 2020), which should be considered when choosing amendments.

Amendments containing S show promise for lowering grain As in rice, but further study is required. There were a limited number of recent studies on the effect of S on As in rice, and they were all pot studies. The response of rice plants to S amendments under field conditions may be different, especially because there are consistent but poorly understood interactions with soil biogeochemistry that are not well simulated in pot studies. Further research to determine all mechanisms of action and behavior on a field-scale is crucial. Due to the general decrease in effectiveness under aerobic conditions and the increase in effectiveness at higher rates of application, S amendments are most effective at moderate to high rates to flooded rice.

#### Biochar and charred or ashed amendments

Biochar and charred/ashed amendments have been investigated for their ability to lower As uptake. These carbon-rich amendments are made from burning or charring organic material, frequently plant material. Biochar is produced by pyrolysis which involves burning organic material at high temperatures, typically from 300 to 700 °C, with little or no oxygen, while chars and ashes are produced by burning organic materials at high temperatures without limiting oxygen (Reed et al., 2017; Tomczyk et al., 2020). The chemical properties of biochar and chars/ashes vary due to the type of organic material used as a substrate (Tomczyk et al., 2020), burning temperature (Linam et al., 2022), and heating rate. Because components within biochar can complex with As oxyanions to render them immobile (Namgay et al., 2010), they can be effective at decreasing As uptake. Biochars and char/ash materials can also provide nutrients such as Si that can compete with As for plant uptake (Penido et al., 2016). Biochar application can be beneficial as it improves soil water retention and crop yield, increases soil organic C, enhances nitrogen use efficiency, and provides a use for crop residue (Lehmann et al., 2006; Novak et al., 2009; A. Zhang et al., 2012). It can also lower GHG emissions, as it is a carbon sink, and decrease the uptake of Cd (Cui et al., 2011; Lehmann et al., 2006).

Additional benefits aside, the response of soil As to biochar and char/ash application remains inconsistent. Biochar or char/ash application lowered total grain As levels by an estimated 18–56% in the rice grain in some studies (Kabir et al., 2021; Leksungnoen et al., 2019; L. Lin et al., 2017; G. Liu et al., 2020; Seyfferth et al., 2019a, 2019b; Seyfferth et al., 2016), but other studies found that they had no impact on total grain As (Limmer et al., 2018a, 2018b; Linam et al., 2022; R. Ma et al., 2014; Qiao et al., 2018; Teasley et al., 2017; Z. Yu et al., 2017a, 2017b). Biochar application can also alter soil pH (A. Zhang et al., 2012), which is not always desirable, and adsorb nitrate and ammonium which could potentially lead to N deficiency (Lehmann et al., 2006). One study found that biochar caused an increase in total grain As when applied at a rate of 20 t ha^−1^ as it raised the soil pH to 9, which caused As oxyanions to be released from sorption by decreasing the number of positively charged sites in soil minerals (Kabir et al., 2021). Other studies at much lower rates of application found that biochar did not affect soil pH (Linam et al., 2022; Seyfferth et al., 2019a, 2019b; Teasley et al., 2017) and was effective at lowering grain As concentrations in some conditions.

Of the studies showing that biochar or char/ash amendment decreased total grain As, some studies determined As speciation in the grain and found that the reduction was due to a 15–50% decrease in iAs (Leksungnoen et al., 2019; L. Lin et al., 2017; Seyfferth et al., 2019a, 2019b; Seyfferth et al., 2016). Of the studies showing no biochar effect on total grain As, the results were more mixed, with some studies seeing a decrease in the ratio of iAs to oAs (Limmer et al., 2018a, 2018b; Z. Yu et al., 2017a, 2017b) and others finding no change in the ratio (Ma et al., 2014; Teasley et al., 2017). Most studies finding decreased total grain As used biochar or char/ash made from rice husk, a Si-rich substrate that raised the amount of Si in the porewater (Leksungnoen et al., 2019; Seyfferth et al., 2016, 2019a, 2019b). Si addition has been shown to inhibit the uptake of iAs by competing with it for transport into the plant, promoting the formation of poorly crystalline Fe plaques on the root surfaces that have the ability to adsorb As, and stimulating As methylating microbes, which can decrease the ratio of iAs to oAs (2019a, 2019b; Dykes et al., 2021; Leksungnoen et al., 2019; Seyfferth et al., 2016).

Further research on drawbacks, optimum substrate, and rate of application is needed before biochar can be recommended as a method for lowering As accumulation in rice. We suggest that if biochar is applied as a soil amendment, the chemical components of the biochar substrate should be considered. If the biochar’s substrate material is contaminated with heavy metals, as some substrates are, applying biochar then increases contaminant levels in the soil and the plants (Lucchini et al., 2014). While biochar can serve as a carbon sink, it can also increase GHG emissions depending on the amount of GHG released during biochar production, the type of biochar, the rate of application, and the properties of the underlying soil (Bu et al., 2022; Zhang et al., 2012; Zimmerman et al., 2011). Biochar may be a good amendment to use when soils have a mix of contaminants, especially Cd, as biochar is better at binding Cd than As (Linam et al., 2021; Qiao et al., 2018). Biochar tends to be negatively charged, which binds more easily to positively charged Cd ions than negatively charged As oxyanions (Qiao et al., 2018).

#### Phosphate

Application of phosphate as a soil amendment can decrease As levels in rice because iAs(V) competes with phosphate for transport into the plant (Abedin et al., 2002; Meharg & Hartley-Whitaker, 2002), but it can also increase As levels in rice by competing with As for sorption sites in the soil, resulting in higher As bioavailability (Bolan et al., 2013). It can also increase the levels of other heavy metals in the soil as rock phosphate often contains Cd (Roberts, 2014). There have also been reports of phosphate application increasing Pb accumulation under some water management conditions (Wu et al., 2021), which can be a concern in mine-contaminated soils with high levels of both As and Pb (Yang et al., 2018). Moreover, iAs(V) is a minor component of the As found in most traditionally flooded rice paddies, so it likely has limited effectiveness under field conditions.

Studies on phosphate application found that some rates of application lowered total grain As 14–79% (Chattopadhyay et al., 2021; Singh et al., 2023), while other rates either had no effect (Irem et al., 2019; Yang et al., 2020) or increased grain As 11–33% (Chattopadhyay et al., 2021; Hossain et al., 2009; Yang et al., 2020, 2021). The response was somewhat rate-dependent as rates of application at or above 50 mg kg^−1^ either increased or had no effect on total grain As (Hossain et al., 2009; Irem et al., 2019; Yang et al., 2020, 2021). However, Chattopadhyay et al. (2021) found that phosphate applied at a rate of 10 mg kg^−1^ caused an increase in total grain As while phosphate applied at rates of 20, 30, and 40 mg kg^−1^ caused a decrease in total grain As, so intermediate rates of application may be preferable. Phosphate amendments were able to lower both oAs and iAs in rice grain but only at intermediate rates of application (Chattopadhyay et al., 2021). At the lowest rate of application, phosphate amendments caused a 50% increase in grain oAs (Chattopadhyay et al., 2021).

The effect of phosphate amendments on total grain As also depended on interaction with soil nutrient status and with other management practices. In soil that was initially P-sufficient, applying phosphate lowered total grain As, but in soil that was initially P-deficient applying phosphate raised total grain As (Dang et al., 2016). The low amount of P in the soil may have caused upregulation of the P transporters that also transport iAs(V) (Dang et al., 2016). Combining phosphate amendments with aerobic irrigation practices also enhanced treatment effectiveness. Phosphate amendments with AWD and fully aerobic irrigation methods lowered grain As up to 66%, a higher reduction than the 17–45% either treatment was able to do alone (Talukder et al., 2012; Wu et al., 2021). Phosphate competes most effectively with iAs(V), which is the form of As that predominates under aerobic conditions (Zhao et al., 2010a, 2010b).

Phosphate amendments can decrease As uptake but should be used carefully. Though phosphate should be applied at a sufficient rate to compete effectively with As, there is evidence that most soils have an optimum rate of application beyond which no additional benefit is provided and a point beyond that at which phosphate application may become actively harmful to the plant (Campos-Soriano et al., 2020) or the environment. Overuse of phosphate results in water pollution as the excess runs off, causing eutrophication and encouraging algae blooms that are detrimental to the environment (Ayoub, 1999) so high rates of application should be discouraged. It would be best to apply phosphate in conjunction with aerobic water management practices for maximum effectiveness for As mitigation.

#### Organic matter

Like biochar, organic matter can immobilize As through interactions between functional groups and As (Bhattacharyya et al., 2003; Roth et al., 2012). Organic matter amendments include composted vegetation, farmyard manure, worm castings, and biogas residue, and as such are readily available to most farmers, often at low cost (Muchovej & Pacovsky, 1997). Different forms of organic matter vary in physicochemical characteristics, including cation exchange capacity and pH, but all forms used as fertilizer are high in essential plant nutrients such as N, P, and K (Muchovej & Pacovsky, 1997). Organic amendments improve soil stability and water retention, encourage the growth of soil microorganisms, and increase plant biomass and yield (Muchovej & Pacovsky, 1997). However, organic amendments are not exclusively beneficial. Depending on the source they can contain heavy metals, pathogens, and other phytotoxic compounds (Alvarenga et al., 2015; Duan et al., 2012). Some forms of organic amendment can increase plant accumulation of As either by containing high amounts of As or by lowering the soil redox potential, which releases Fe-mineral bound As and thus increases plant-availability (Norton et al., 2013).

The response of As accumulation to organic amendments in this review was mixed. In some studies, organic amendments lowered total grain As by 8–38% (Irem et al., 2019; Li et al., 2023; Sengupta et al., 2021), while in others they had no impact (Alvarenga et al., 2022; Duan et al., 2012; Li et al., 2022a, 2022b; Ma et al., 2014; Tang et al., 2021) or raised total grain As (Islam et al., 2020; Norton et al., 2013; Xiao et al., 2017). In some cases, this effect was dependent on the rate of application as in T. Li et al. (2023), where the lowest rate of application caused a decrease in total grain As while the higher rates of application increased grain As. The amendment in this case had a pH of 8.3, which raised the pH of the soil at higher rates of application, resulting in the mobilization of As (Li et al., 2023). In other cases it was site-dependent, for example when organic amendments lowered total grain As for rice grown in soil from one site but not for rice grown in soil from two other sites (Codling & Chen, 2017).

The combination of AWD or sprinkler irrigation with organic matter amendments was also somewhat ambiguous, with one study finding the combination lowered grain total As up to 65% compared to the 16–26% reduction of either method alone (Sengupta et al., 2021), while others found that the decrease in grain As was driven solely by the irrigation method (Alvarenga et al., 2022; Islam et al., 2020). However, Alvarenga et al. (2022) found that adding organic matter lowered the ratio of iAs to oAs in the grain and offset the increase in grain Cd caused by aerobic irrigation.

Because organic amendments do not consistently decrease grain As levels and can sometimes increase it, they are not ideal when applied solely to limit grain As concentrations. Due to the widespread use of organic matter as fertilizer, it would be better to evaluate the mechanisms that control As uptake and to tailor organic amendments to the biogeochemistry of sites rather than limit its use. Organic amendments should be tested for toxic elements, and forms of amendment that contain high levels of As or other toxic elements should be avoided. The pH should also be evaluated as some organic amendments have a high pH and should only be applied at high rates in acidic soils.

#### Combination amendments

Different amendments for mitigating As contamination in rice can be applied simultaneously. Combination amendments are used to improve the effectiveness of a single treatment alone or, in some cases, to mitigate the increase in grain Cd levels that may accompany treatment-induced decreases in grain As. This application makes combination treatments ideal for soils with multiple toxic elements of concern.

Some combination amendments are made of components with different mechanisms of action for preventing grain As accumulation. Lin et al. (2017) used a combination of biochar, Mn oxides, and Fe compounds in the form of either ferric nitrate or ferrous sulfate to lower grain total As by up to 77%, more effectively than biochar applied by itself, and demonstrated a maximum decrease of 42%. Another study similarly combined Mn oxides and biochar to lower grain total As 14–18% where biochar alone either increased grain total As or had no impact (Z. Yu et al., 2017a, 2017b). The Fe compounds encouraged the formation of root plaques that adsorbed As, while the biochar immobilized As in the soil and the Mn oxides facilitated the oxidation of iAs(III) to the less mobile iAs(V) (Lin et al., 2017; Yu et al., 2017a, 2017b). Other combination amendments are chosen for their ability to decrease the uptake of both As and Cd, two toxic elements that often co-occur in the soil. Biochar, which has a stronger ability to immobilize Cd than As, has been used in combination with Fe compounds (Qiao et al., 2018; Zhou et al., 2022), Mn compounds (S. Zhou et al., 2022), and phosphate (Gu et al., 2019), all of which have the ability to limit As uptake. Biochar has also been used in combination with other Cd-limiting components like sepiolite, a clay mineral that adsorbs Cd in the soil (S. Zhou et al., 2022), and zeolite, an aluminosilicate mineral that can adsorb Cd (Gu et al., 2019). These combinations of biochar and other amendments were able to lower grain total As up to 61% while also lowering grain Cd up to 93% (Gu et al., 2019; Qiao et al., 2018; Zhou et al., 2022).

### Postharvest management

In addition to agronomic practices that limit As accumulation in the rice grain, there are ways of processing and managing rice post-harvest that reduce consumer As ingestion. One method is simply to mill the grain from brown rice to white rice, removing the bran from the grain. Bran accumulates more As than the rest of the grain (Meharg et al., 2008; Sun et al., 2008), and its removal can reduce the grain As concentrations by 26–39% (Fontanella et al., 2021; Naito et al., 2015). Unfortunately, some of the nutritional benefits of brown rice are then lost, as the bran is higher in vitamins, micronutrients (Fe, Zn), protein, and fiber (Babu et al., 2009). A diet higher in micronutrients leads to lower bioaccessiblity of a given toxic element like As to the human diet. The benefits of eating brown rice outweigh the As risks in adults who have a varied diet, do not consume rice with grain iAs levels higher than 0.1 mg kg^−1^, and do not ingest additional As in drinking water (Menon et al., 2022). The risks of As ingestion via rice are higher for infants and young children, who have a lower body weight and for whom rice may compose a larger portion of the diet via rice-based cereals and snacks (Jara & Winter, 2014; Signes-Pastor et al., 2016).

The level of As in cooked rice can be changed by a modified form of parboiling. In rice processing, parboiling means soaking and steaming unhusked rice to decrease grain breakage during milling and to improve storage quality (Araullo et al., 1976). The resulting rice is called converted rice and it is both less susceptible to insect damage and has higher nutrient levels than unconverted rice (Araullo et al., 1976). Converted rice is typically higher in As than raw rice, partially because the steaming process results in the transfer of As from the husk to the grain and because it retains more of the bran than raw rice once milled (Araullo et al., 1976; Fontanella et al., 2021). However, a recent study has shown that parboiling the rice after husking rather than before results in As leaching from the grain into the parboiling water, lowering the total iAs in the final product by 25% (H. Rahman et al., 2019). Similarly, home cooking methods that involve briefly parboiling rice before cooking and discarding the parboiling water lowered the As up to 83% in the cooked grain, as did fully cooking the rice in excess water and draining it before serving (Atiaga et al., 2020; Chowdhury et al., 2020; Menon et al., 2021; Mwale et al., 2018). Rinsing or soaking the rice in several changes of water before cooking also lowered As up to 50% in cooked white rice (Atiaga et al., 2020; Chowdhury et al., 2020; Menon et al., 2021). Only parboiling was able to lower As in cooked brown rice because the As bound to the bran layer resists removal without the application of heat (Menon et al., 2021). Parboiling on an industrial scale requires more energy and labor than just milling raw rice, but rinsing, soaking, and modifications in cooking require minor amounts of water and energy and are viable methods of reducing As for home cooks.

## Stakeholder feedback

The stakeholder interviews successfully allowed conversations with growers representing four farms, four supply chain participants (representing mills or grain dealers), and one academic researcher in the rice field. Most participants were from Arkansas or its neighboring states and represented practices common in the U.S.’s mid-South production region. Some key themes emerged, foremost is that there is very little being done currently to extensively monitor or deliberately decrease As in U.S. rice beyond occasional grab sampling at the mills to monitor As levels. The interviewees all noted that they would alter their practices if changes were mandated, deemed necessary, and incentivized. Many interviewees expressed concern with their lack of knowledge of the hazards of As in the grain. They wanted to know what the regulatory limits are, what likely changes are coming, why a change may be important, and what they can do. Thus, we suggest that any mandated changes should be concurrent with education and awareness campaigns that include tight links to university extension or U.S. Natural Resources Conservation Services (NRCS) outreach professionals, or their equivalent in other nations. Four other themes were consistently emphasized by the respondents, and are highlighted below, along with suggestions for where to prioritize research funding:

### Data needs

Each stakeholder expressed a general concern regarding As in rice, particularly to protect infants and young children, but most agreed that more information was needed. That is, they would help to reduce grain As levels if asked to, but often they did not know whether there was a need to reduce them, what the evidence basis was, and what opportunities their part of the rice production system allowed for reducing grain As levels. They recognized the difficulty of performing a scientifically controlled study on the impacts of As levels in rice on health outcomes in infants and young children. It was also noted that some of our current understanding of health impacts caused by As comes from data on contaminated water that have been extrapolated for application in diet studies.

### Expenses

There are significant system changes needed to implement grain identity preservation focused on low-As grain and growth conditions. Challenges include reliably sourcing low-As rice grain, the need for additional storage and transportation in and between farms and the mill, and advertising low-As rice. There are currently few financial incentives and little support to make these changes. Changes to the milling processes such as increasing the use of parboiling would require significant investment to re-fit or build new plants. Ultimately, the expenses of moving toward zero-As rice would likely be higher at the mill side and lower on the farm side, depending on the management route taken.

### Arsenic testing facilities

Current rice grain testing takes between one week and two months for total As analysis, and it can take even longer for speciation of the iAs fraction. These analyses can cost approximately $200 per sample at commercial laboratories where the mill members regularly send samples to test for iAs. Many respondents indicated that a faster, cheaper testing solution could help farmers implement agronomic changes from one season to the next, assist with sourcing rice for more sensitive populations, and accelerate the research process that still needs to uncover a more mechanistic understanding to link soil and grain As levels. Investment in accurate, high-throughput testing would also enable farmers who grow low-As rice to contract with mills to provide low-As rice and rice products intended for sensitive populations, a pipeline that currently does not exist.

Our recommendation is to create regional, public-sector laboratories, perhaps run by USDA or/and land-grant institutions, for high-throughput As speciation analysis that could facilitate creating a pipeline for low-As rice streams. Such a system could be modeled after laboratories already providing such services for microbiological food safety concerns. Developing a handheld kit or sensor that a farmer could use just before or during harvest would be valuable to maintain any low-As contracts, but this technology does not currently exist.

### Lack of production scale evidence

The respondents noted that many mitigation methods have been tested in small-scale studies but there is not an abundance of data from the critical production scale of commercial farms and high-volume millers and processers. For example, many of the changes in irrigation strategy towards aerobic production environments are possible in some places but are more difficult in others: independently controlled water supplies are particularly important. Production environments in California and Texas often rely on canal water that is ordered in advance, so it would likely be difficult to transition to AWD and to time the drying events with water supply availability. In those regions, switching to furrow irrigation may be more practical than switching to AWD. Farmers in these regions could also perform a type of AWD where 20–30% of the topographically higher part of the field can receive drying events even if the lower fields cannot be adequately drained. Even when AWD is intended, the soil can remain inundated if rainfall arrives during an intended drying period. In either case, more field-level research should be performed to balance decreasing grain As levels with the risk of yield reductions due to a drier soil environment. Several farmers mentioned the risk of weed growth associated with AWD as a prohibiting factor. There are also farm infrastructure barriers to implementing AWD due to the layout of the pump and canal distribution networks.

Most respondents assumed that soil or foliar amendments would have only incremental material costs and could likely be implemented in tandem with other applications or using existing technologies. However, the additional labor needs and uncertainty in the amendment application (e.g., incorporation method, rates, timing, chemical costs, etc.) need to be incentivized financially and encouraged by the mills. Low bulk density additions such as rice husk would require more time for application given the number of trips required to apply them, which adds to labor and tractor diesel costs. The farmers noted that in some years, weather limits the amount of time where field preparation work, such as amendment incorporation, can be performed. There are other time-based concerns, as the farmers have the least available time during planting and harvest periods, so additional management requirements during those periods would be particularly difficult or expensive to implement. Additionally, for widespread implementation of changes, the respondents noted that more change-averse farmers would likely require higher financial incentives to change long-standing agronomic practices. Similarly, any documentation requirements (e.g., for achieving an AWD drying event to a certain depth) may require additional labor or technology and the cost of that monitoring or reporting should be included in incentive programs.

There is a suite of research needs, including structuring incentive programs for mitigation efforts. We also need to assess enabling structures such as education and grain testing, mill- or farm-side storage, and ways to maintain identity preservation. One respondent noted that a large, systems-level federally funded research project, with support from the rice industry and its many stakeholders, would be beneficial. Such an initiative could coordinate with the different rice-growing states and balance As concentration targets with other needs, including water use, greenhouse gas emissions, and farm and mill economics.

## Discussion

Upon reviewing treatments for lowering grain As in rice, some methods were more effective than others (Table 2). A treatment was considered highly effective if the majority of studies in this review concerning that treatment demonstrated lower grain total As, moderately effective if roughly half of the studies demonstrated lower grain total As, and of low effectiveness if fewer than half of the studies demonstrated lower grain total As. The most effective treatments were cultivar selection, irrigation management, postharvest management, and the application of Se or Si amendments. All the most effective treatments had a median low of less than 0.4 mg kg^−1^ for grain total As in both brown and white rice, and all but the Si amendment treatment had a median low below 0.2 mg kg^−1^ (Table 3). Treatments with moderate effectiveness. such as biochar, Fe, or S amendments, are worthy of consideration; however, their implementation should be balanced against cost, difficulty of application, and any drawbacks or conditions of effectiveness, such as the potential for Fe amendments and biochar to contain heavy metals depending on the substrate material. Treatments with low effectiveness, such as organic matter or phosphate amendments, either did not lower or caused an increase in grain total As for the majority of studies in this review and are not recommended.

**Table 2:** An overview of all methods for decreasing grain total As in rice covered in this review

Method and number of studies in review	Affects plant			Affects soil		Grain total As % reduction range	Potential increase in heavy metals	Effectiveness
	Limits uptake of As	Inhibits internal transport of As	Improves growth which dilutes the amount of As in plant tissue	Immobilizes As in the soil	Affects redox potential decreasing plant-available As			
Cultivar Selection (n = 18)	✓	✓	✓	✓	✓	4–100	✓	High
Irrigation Management (n = 31)	–	–	–	✓	✓	10–98	✓	High
Silicon Amendment (n = 13)	✓	✓	✓	–	–	7–78	✓	High
Selenium Amendment (n = 10)	✓	✓	✓	–	–	7–100	–	High
Iron Amendment (n = 14)	✓	–	✓	✓	–	13–92	✓	Moderate
Sulfur Amendment (n = 7)	✓	✓	–	✓	–	27–72	–	Moderate
Biochar Amendment (n = 12)	–	–	✓	✓	–	18–56	✓	Moderate
Phosphate Amendment (n = 9)	✓	–	✓	–	–	14–79	✓	Low
Organic Matter Amendment (n = 12)	–	–	✓	✓	✓	8–38	✓	Low
Combination Treatments (n = 10)	✓	✓	✓	✓	✓	13–100	✓	Varied
Postharvest Management (n = 7)	–	–	–	–	–	12–83	–	High

This table includes the mechanism of action for each treatment, an estimated range of percent grain As reduction, whether the treatment has the potential to increase heavy metal accumulation rather than decrease it, and general effectiveness. Combination treatments refer both to combination amendments and the concurrent application of both amendments and irrigation management. Some studies covered multiple methods

**Table 3:** A comparison of the lowest total As (tAs) values found in brown and white rice for the most effective treatments in the review

Treatment	Number of studies, brown rice	Lowest tAs in brown rice (mg kg^−1^)	Median lowest tAs in brown rice (mg kg^−1^)	Number of Studies, White Rice	Lowest tAs in white rice (mg kg^−1^)	Median lowest tAs in white rice (mg kg^−1^)
Cultivar Selection (Field studies)	10	0.011* (Sandhi et al., 2017)	0.11	1	0.19 (Pillai et al., 2010)	0.19
Irrigation Management (Field studies)	16	0.0013 (Spanu et al., 2012)	0.13	2	0.035* (Carrijo et al., 2018)	0.083
Postharvest Management	4	0.04 (Naito et al., 2015)	0.1	6	0.018* (Menon et al., 2020)	0.025
Si Amendment (Field studies)	1	0.28* (Wang et al., 2016)	0.28	1	0.20* (Limmer et al., 2018a, 2018b)	0.20
Se Amendment (Pot studies)	6	0.054 (Paniz et al., 2023)	0.17	1	0.23* (Pokhrel et al., 2020)	0.23

The * indicates that the value was not directly stated in the source and was estimated from figure(s). We prioritized field studies over pot studies for this summary; our review only found pot studies for Se amendments, so those are shown. Most studies did not measure As levels for both brown and white rice, hence the As for white rice exceeding brown rice in some cases. White rice always accumulated less As than brown rice from the same study. Studies that did not state whether the results were for brown or white rice were excluded from this comparison

When considering As mitigation treatments, it is important to distinguish between scientifically effective and practically achievable. Due to the high cost of modifying milling plants to increase the usage of parboiling and the commercial demand for brown rice as a more nutrient-dense alternative to white rice, the balance of evidence points more towards on-farm mitigation strategies than postharvest management strategies. Cultivar selection, while highly effective, may or may not be practical for most farmers. Due to high rates of cultivar turnover and the necessity of choosing cultivars for traits other than As accumulation such as yield and disease-resistance, the implementation may be complicated. The introduction of As testing into commercial cultivar performance trials would be beneficial, but deliberate breeding for low-As cultivars is unlikely to be feasible except perhaps in areas where As contamination is very high. Se amendments show great promise in small-scale studies but need evaluation on a full field scale before they can be recommended and may have unwanted environmental impacts.

Of all the highly effective potential treatments, Si amendments and irrigation management with aerobic periods are probably the most universally applicable. Si amendments are highly effective, especially in combination with aerobic irrigation treatment, and not difficult to source. However, they may be time-consuming or difficult to apply, depending on the form, and that should be factored in when considering an economic analysis. Moreover, they may be more effective for rice grown in highly weathered, Si-deficient soil than those in the U.S. where rice soils are clay-rich and have more plant-available Si. Fully aerobic and AWD irrigation can be difficult to implement in some fields depending on the grade of the field, weed and disease pressure, and the management of the water supply, but furrow irrigation is a viable alternative in situations where AWD is not possible. Irrigation methods with aerobic periods are not without drawbacks, as they carry a higher risk of reduced yield and increased grain Cd levels than flooded irrigation treatment, but these risks can be minimized by avoiding fully aerobic or rainfed irrigation.

A common limitation of all treatments in this review is the relative lack of field studies compared to pot studies. Evaluation on a field-scale is vital to determining whether results in a pot study are comparable to results on a commercial level. Furthermore, the effect of many treatments was dependent on the other management practices and the soil and environmental conditions at each site. The baseline soil As levels in this review varied widely by study, with some sites at background concentrations (e.g., 5 mg kg^−1^) and others heavily contaminated (> 25 mg kg^−1^). The best recommendation for heavily contaminated soils might be to encourage remediation before utilizing that field for growing rice, and a field with background soil As concentrations may not need intervention. It is clear from the stakeholder interviews that the biggest barriers are practicality, time, and cost. For some mitigation strategies, implementation may only be possible with significant incentivization which limit intervention to areas with the greatest need for As reduction. This focus necessitates cheaper and faster testing for total As and iAs in rice grains so that stakeholders can determine what interventions are necessary and for what locations.

## Conclusion

Methods to lower As accumulation in rice can be applied at multiple rice production stages, during the growing season, and following harvest. The best method for a particular field may need to be tailored to the management of that field and the concerns of the producers.

Among the mitigation practices applied during the growing season, soil amendments such as Si and irrigation management with aerobic periods have proven to be viable for decreasing rice grain As concentrations and can be broadly applied. There is a need for further field-scale studies for all methods applied prior to harvest. Among the mitigation practices applied after harvest, milling brown rice to white rice, and parboiling after husking have proven effective, but are likely costly and difficult to implement on an industrial scale. Educating home cooks on the benefits of rinsing, soaking, and parboiling may be more easily implemented.

Interviews with stakeholders revealed that they are amenable to implementing As mitigation methods if deemed necessary, but that incentivization would likely be required to achieve mass application. The separation of rice sources with differing levels of grain As is possible but would be difficult to manage logistically and would require cheaper and quicker grain As testing to be made practical.

## Supplementary Information

Below is the link to the electronic supplementary material.

Supplementary file1 (XLSX 55 kb)

## Data Availability

No datasets were generated or analysed during the current study. A spreadsheet containing arsenic data from the reviewed literature is provided as supplementary material to this article.
